# A systematic review of factors that shape implementation of mass drug administration for lymphatic filariasis in sub-Saharan Africa

**DOI:** 10.1186/s12889-017-4414-5

**Published:** 2017-05-22

**Authors:** Adam Silumbwe, Joseph Mumba Zulu, Hikabasa Halwindi, Choolwe Jacobs, Jessy Zgambo, Rosalia Dambe, Mumbi Chola, Gershom Chongwe, Charles Michelo

**Affiliations:** 1 Department of Health Policy and Management, School of Public Health, University of Zambia, PO Box 50110, Lusaka, Zambia; 2 Department of Health Promotion and Education, School of Public Health, University of Zambia, PO Box 50110, Lusaka, Zambia; 3 Department of Environmental Health, School of Public Health, University of Zambia, PO Box 50110, Lusaka, Zambia; 4 Department of Epidemiology and Biostatistics, School of Public Health, University of Zambia, PO Box 50110, Lusaka, Zambia

**Keywords:** Lymphatic filariasis, Mass drug administration, Implementation, Barriers and facilitators, Sub-Saharan Africa

## Abstract

**Background:**

Understanding factors surrounding the implementation process of mass drug administration for lymphatic filariasis (MDA for LF) elimination programmes is critical for successful implementation of similar interventions. The sub-Saharan Africa (SSA) region records the second highest prevalence of the disease and subsequently several countries have initiated and implemented MDA for LF. Systematic reviews have largely focused on factors that affect coverage and compliance, with less attention on the implementation of MDA for LF activities. This review therefore seeks to document facilitators and barriers to implementation of MDA for LF in sub-Saharan Africa.

**Methods:**

A systematic search of databases PubMed, Science Direct and Google Scholar was conducted. English peer-reviewed publications focusing on implementation of MDA for LF from 2000 to 2016 were considered for analysis. Using thematic analysis, we synthesized the final 18 articles to identify key facilitators and barriers to MDA for LF programme implementation.

**Results:**

The main factors facilitating implementation of MDA for LF programmes were awareness creation through innovative community health education programmes, creation of partnerships and collaborations, integration with existing programmes, creation of morbidity management programmes, motivation of community drug distributors (CDDs) through incentives and training, and management of adverse effects. Barriers to implementation included the lack of geographical demarcations and unregistered migrations into rapidly urbanizing areas, major disease outbreaks like the Ebola virus disease in West Africa, delayed drug deliveries at both country and community levels, inappropriate drug delivery strategies, limited number of drug distributors and the large number of households allocated for drug distribution.

**Conclusion:**

Mass drug administration for lymphatic filariasis elimination programmes should design their implementation strategies differently based on specific contextual factors to improve implementation outcomes. Successfully achieving this requires undertaking formative research on the possible constraining and inhibiting factors, and incorporating the findings in the design and implementation of MDA for LF.

## Background

Globally, over 947 million people are at risk of infection with lymphatic filariasis (LF) [1] and an estimated 67.88 million are infected, with as much as 36 million people disfigured and incapacitated by its resultant chronic conditions [2]. According to the World Health Organization (WHO), LF accounts for at least 2.8 million disability adjusted years (DALYs) not including significant co-morbidity of mental illness commonly experienced by patients and their caregivers [1, 3]. This disease affects the poorest populations in society, particularly those living in areas with poor water, sanitation and housing, causing permanent disfigurement, reduced productivity and social stigma [4]. The most common chronic manifestations of LF are lymphedema (swelling of skin), elephantiasis (swelling of limbs) and hydrocele (swelling of genital organs) [5].

South-East Asia and sub-Saharan Africa (SSA) account for about 94% of the LF global disease burden [6]. The SSA region is estimated to have 409.7 million people from 35 endemic countries at risk of infection [7], which is about 32% of the LF global disease burden [2]. LF is associated with massive economic losses in SSA, impairing economic activity of up to 88% in infected people and causes almost US$1 billion in annual productivity losses, mostly resulting from the disability linked to hydrocele in men [8, 9].

In response to the global burden, the WHO formed the Global Programme to Eliminate Lymphatic Filariasis (GPELF) in 2000 [10]. The GPELF strategy has been to promote large scale mass drug administration (MDA) in endemic areas with annual doses of albendazole, ivermectin or diethylcarbamazine citrate (DEC) and provision of minimum care to every person with associated LF chronic manifestations [11]. The core objective of MDA for LF is to reduce microfilariae levels in human populations in order to interrupt the transmission cycle between mosquitoes and humans. A minimum annual MDA for LF coverage of >65% of the population at risk is recommended by the WHO for 4–6 years [12]. However, this is usually dependent on the microfilariae baseline prevalence in the population at risk and other factors determining transmission [13].

Over the years, many countries have implemented MDA for LF campaigns, successfully reducing the prevalence levels of the microfilariae in endemic populations. Between 2000 and 2015, the WHO reported that more than 6.2 billion doses of treatment were administered to more than 830 million people in 64 endemic countries, reducing the transmission risk by 45% [6].

Despite these positive global achievements in treatment provision, MDA for LF programmes continue to face numerous challenges that result into low treatment coverage levels and non-compliance by the communities [12, 14–19]. Programmatic challenges include; sustaining timely distributions of drugs, establishing accurate monitoring and evaluation systems by the communities, increasing involvement of the local communities and engaging in effective advocacy for continued MDA for LF support [12]. Lahariya et al. [20] notes that MDA for LF programmes seem to focus more on tablet distribution than the major implementation questions such as health education, side effects, morbidity management and logistics. Similarly, Kisoka et al. [21] also reports that problems of low compliance to MDA for LF are more provider-initiated than by individual recipients’ perceptions and practices.

Several publications in SSA have highlighted implementation challenges in MDA for LF programmes [21–25]. However, systematic documentation of this information still remains lacking. Understanding the factors shaping implementation is critical to strengthening future MDA for LF campaigns. This review therefore, aims at systematically documenting the barriers and facilitators to implementation of MDA for LF in SSA.

## Methods

### Search strategy

A systematic search of three databases PubMed, Science Direct and Google Scholar was conducted between December 2015 and May 2016, to document facilitators and barriers to implementation of MDA for LF. We also searched references of retrieved articles to identify further literature. The key search terms included: “community directed treatment,” OR “community participation,” OR “community drug distributors,” OR “acceptability” OR “compliance” OR “Coverage” OR “implementation” AND “lymphatic filariasis” AND “mass drug administration.” AND “sub-Saharan Africa.”

### Inclusion and exclusion criteria

**Fig. 1:**
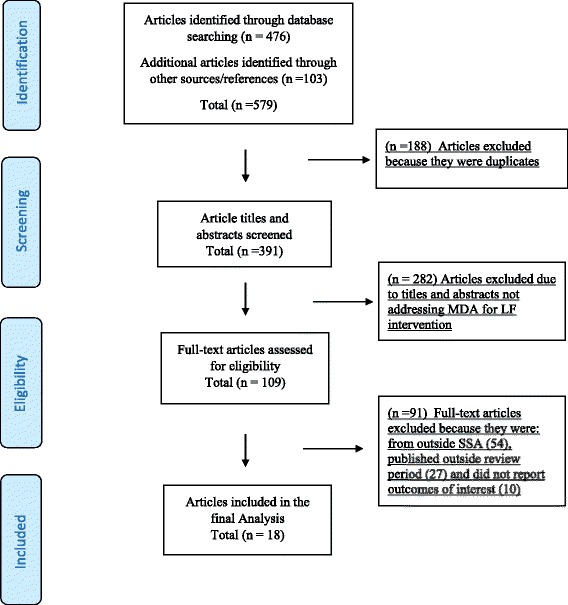
PRISMA flow diagram

The search was limited to English peer-reviewed publications for LF MDA programmes implemented in the sub-Saharan Africa region. Only publications from 2000 to 2016 were included, as this marked the period of heightened international efforts to eliminate LF as a public health problem. Studies that illustrated the implementation processes, highlighted strategic lessons learnt from evaluations and documented national programme successes were included. Studies were included if they assessed the following implementation outcomes; (i) Treatment coverage/compliance, defined as the proportion of individuals, expressed as percentage of the target population who received and swallowed a drug or combination of drugs [26]; (ii) Program sustainability, referred to the process of maintaining or institutionalising innovation use, capacity and benefits [27, 28]; (iii) Successful implementation referred to perceptions among implementation stakeholders (both the provider and community) that a given treatment, service, practice, or innovation is agreeable, palatable, or satisfactory with their needs [28]; and (iv) Community participation, defined as the involvement of the community in programme design implementation and evaluation [29]. Publications were excluded if they did not address the MDA for LF intervention, were from outside the SSA region, conducted before the year 2000 and did not report any of the MDA for LF implementation outcomes as defined by the review (Fig 1).

### Study selection and quality assessment

The study selection was guided by the PRISMA guidelines by Moher et al. [30]. The search resulted in 579 articles, of which 188 duplicates were excluded. The remaining 391 titles and abstracts were screened and 282 articles were excluded for not addressing the MDA for LF intervention. A total of 109 articles were then considered for full text reading, of which 91 papers were eliminated because they were not from sub-Saharan Africa, published outside the review period and did not report the outcomes of interest. The final 18 articles have been reported in this systematic review (Fig 1). The quality of these studies was assessed using the critical appraisal skills programme [31]. Data was extracted onto a data extraction form created in Microsoft excel to asses information on key study aspects such as the findings, designs, sample, data collection, analysis, reporting and ethics.

### Data analysis and synthesis

Data from the selected articles was analyzed using thematic analysis technique [32], in NVivo 10 software (QSR international, Melbourne, Australia). This technique enables identification and exploration of themes and relationships within the coded data. A code list was developed comprising of structural/broad themes, which were iteratively agreed upon by the research team members after preliminary reading of abstracts and later modified to accommodate emergent themes. The code-list was then imported to NVivo and data from the included articles was coded in the respective nodes. Coding was conducted by two separate researchers including the principal investigator to allow for inter-coder reliability tests. Where there were discrepancies, the researchers met to discuss and reach consensus on how to code the information. Code reports from the coding activity allowed for identification of context specific factors shaping implementation of MDA for LF (Table 2).

## Results

### Study characteristics

A total of 18 sub-Saharan African articles were included in the final analysis. These articles were from the following countries; Nigeria (*n* = 2), Sierra Leone (*n* = 2), Ghana (*n* = 2), Togo (*n* = 1), Liberia (*n* = 1), Mali (*n* = 1), Kenya (*n* = 4) and Tanzania (*n* = 5). Table 1, provides a summary of all the included studies; the MDA for LF implementation period, the study sample characteristics and context (setting and area of the country where MDA for LF was implemented), study objectives, major findings and implementation outcomes. Eleven of the studies had qualitative (*n* = 3), quantitative (*n* = 4) and mixed methods (*n* = 4) designs. Six of the studies were programme reports (*n* = 6) and one was an evaluation (*n* = 1). Table 2, gives details of the identified barriers and facilitators to implementation of MDA for LF, whilst Table 3 tries to quantify some of the recurrent approaches used to improve MDA for LF implementation in the reviewed studies.

**Table 1:** Characteristics of included studies

Author/Country/MDA for LF period	Study type	Sample characteristics/ Context (setting and areas where MDA for LF was implemented)	Study objectives	Study findings	Outcomes
1. Kisoka et al., 2016 [44]/Tanzania/May and August 2011	Qualitative study	Interviews with 21 CDDs, 11 community leaders, 6 religious leaders and 18 FGDs with community members representing adults and adolescents. (Urban and Rural settings) Morogoro and Lindi regions.	To gain insight into targeted community members’ perceptions and experiences of LF, the drugs distributed and the phenomenon of MDA, so as to indicate ways of improving the intervention and the interaction between populations and the intervention for future campaigns.	Investment in appropriate dissemination of accurate and timely MDA for LF information is essential for guaranteeing community support for the programme.	Successful MDA for LF implementation and community participation
2. Bogus et al., 2015 [33]/Liberia/June 2013	Cross sectional study	Interviewed 140 community leaders from 32 villages. (Rural setting) Lofa County, Kolahun District.	To assess community leaders’ knowledge and attitudes regarding resumption of MDA for NTDs after the Ebola virus disease epidemic (EVD).	Shift in national health systems priorities regarding funding, research and development due to EVD. Temporally halt to all MDA for LF activities in EVD affected areas. Fears in the community that EVD and MDA might be linked, hence affecting compliance/coverage	Programme sustainability, community participation and coverage
3. Kisoka et al., 2014 [21]/Tanzania/May and August 2011	Cross sectional household survey	Data was collected from 3279 adults above 15 years of age. (Urban and Rural settings) Lindi and Morogoro regions.	To assess, through household questionnaires, the associations between selected predictors and individual drug uptake shortly after the implementation of MDA in two rural and two urban Districts in Tanzania	Drug uptake relied more on easily modifiable provider-related factors than on individual perceptions and practices in the target population. Motivation of drug distributors to visit all households (repeatedly when residents are absent) are likely to have considerable potential for increasing drug uptake.	Coverage
4. Madon et al., 2014 [45]/Tanzania/not stated	Qualitative study	15 key informants interviews with Voluntary health workers (VHWs), village leaders and health officials. 4 FGDs with VHWs. 4 FGDs with village health committee members. (Rural setting) Pwani region, Mukurunga district.	To relate the conceptualization of mobile telephony in the health sector to the NTD Control programmes in Tanzania	Providing mobile phones to VHWs helped to increase the efficiency of their routine NTD work, boosting motivation and self-esteem.	Community participation
5. Njomo et al., 2014 [25]/ Kenya/May 2011 and October 2012	Mixed methods	Quantitative data collected from 947 household heads. Qualitative data; 12 FGDs with single sex adult and youth male and female groups. 3 FDGs with CDDs. 40 IDIs with opinion leaders and health personnel. (Urban setting) Malindi district.	To identify, design and test strategies that could be used to develop guidelines for achieving high treatment coverage in an urban setting and to identify possible pitfalls that could be a hindrance to achieving high treatment coverage in such urban settings.	Activities identified to improve Urban MDA for LF coverage: adequate engagement of key health systems and community personnel, at all stages of the programme. Use of appropriate, innovative context specific strategies to create awareness in Urban settings. Employ appropriate drug distribution strategies.	Coverage and programme sustainability
6. Offei et al., 2014 [38]/Ghana/ 2012	Cross sectional household survey	Data collected from 384 household heads or any responsible adult above 18 years. (Rural setting) Ahanta-West District.	To explore the level of compliance to the LF programme by the people of Ahanta West District and also estimate coverage during the 2012 MDA programme year.	Improved health education focusing on the safety of drugs and the importance of MDA needs to be undertaken before and during the drug distribution exercises to improve and sustain uptake.	Coverage/﻿compliance
7. Sodahlon et al., 2013 [39]/Togo/2000–2009	LF programme report	National	To describe the elements that proved successful in the national strategy to address LF in Togo.	Identified various factors required for national LF programme success: Sustained political commitment, integration with existing interventions, innovative resource mobilization in environment totally lacking resources and building of very strong partnerships (internal and external)	Successful implementation, programme sustainability and coverage
8. Dembele et al., 2012 [37]/Mali/2005–2011	Integrated NTD control programme report	National	To report on the progress made by the integrated national NTD control programme in Mali, drawing from objectives achieved, documented experiences and pertinent lessons learned of the program from 2007 to 2011, and focusing on only aspects of integrated MDA activities.	For the long-term sustainability, NTD programmes require to be integrated into primary healthcare systems at local level. Delays in drugs reaching the country resulted in MDA postponement, which not only increased the difficulty in the campaign but also minimized the impact of MDA. Local collaborations with the African Programme for Onchocerciasis Control (APOC) and the Onchocerciasis Control Programme (OCP) was essential to sustaining the Mali NTD integration programme. Achieved Improved national geographical coverage achieved for LF from 25% in 2006 to 100% in 2009.	Community participation, programme sustainability and coverage
9. Hodges et al., 2012 [23]/Sierra Leone/June 2010–2011	Programme evaluation	11,824 participants interviewed in the end process evaluation of hard to reach (HTR) sites. (Urban and rural settings) 12 districts and 4 large towns from southern, eastern and northern provinces.	To identify the challenges to effective mass drug administration implementation for LF and the corrective measures taken.	Challenges affecting MDA for implementation included: late country delivery of ivermectin, the availability and motivation of unpaid CHVs, remuneration for CHWs, rapid urbanization and employment seeking population migrations. ‘In process’ monitoring ensured modifications of LF MDA were made in a timely manner to ensure effective coverage was finally attained in HTR locations.	Community participation and Coverage
10. Njomo1 et al., 2012 [24]/Kenya/December 2008	Mixed- method study	Quantitative data: 965 household heads or adult representatives. Qualitative data: IDIs with 80 LF patients, 80 opinion leaders and 15 CDDs. 16 FGDs with single sex-adults and youths, stratified in males and females (Rural setting) Kwale and Malindi districts.	To determine the role of personal opinions and experiences in compliance with MDA for LF.	Drug distribution methods influence compliance to MDA for LF. Lack of perceived benefits of MDA for LF and risk perception contribute to low compliance. Side effects experiences contribute to low compliance	Coverage/﻿compliance
11. Njomo et al., 2012 [35]/Kenya/2008	Qualitative study	15 CDDs, 80 opinion leaders, 80 LF patients, 5 health personnel, 4 LF coordinators and the national programme managers were interviewed. 16 FGDs were conducted with single-sex adult and youth male and female groups. (Rural and urban settings) Kwale and Malindi districts.	To Identify factors associated with CDDs’ motivation and their influence on community compliance to MDA for LF treatment with a view of suggesting mitigating measures.	Factors that influence CDDs’ motivation were: higher education level, trust and familiarity with community members, being trained on LF and an innate desire to help their communities.	Community participation and Programme sustainability
12. Richards et al., 2011 [36]/Nigeria/not stated	LF programme report	Sample was not stated (Rural and Urban setting) Plateau and Nasarawa states.	To report on our 12-year effort to eliminate LF in Plateau and Nasarawa states, which was the first LF elimination effort to be launched in Nigeria.	MDA for LF treatment in urban areas cannot rely on community volunteers and traditional leadership structures. In urban areas, rather than house to house, treatments were organized in central locations that served as distribution posts, commonly near a neighborhood church, mosque, health clinic or hospital	Community participation and coverage
13. Hodges el al, 2010 [22]/Sierra Leone/2010	Cross sectional study	9249 participants were interviewed (Urban and rural setting). Freetown.	To report the implementation strategy, social mobilization, the high coverage achieved in the urban western area and rural western area of Freetown, and the relative cost needed for each person treated during an MDA for LF﻿.	Key elements of success for social mobilization and implementation strategy (use of pretested IEC materials including FAQs, radio phone-ins, mobile texts, expert contact and government key stakeholder buy-in). Describes the independent monitoring used to estimate final coverage in this urban/non-rural setting where the current population size is uncertain. Suggests an implementation strategy and independent monitoring tool that could be useful in similar, rapidly growing cities implementing lymphatic filariasis elimination programmes	Community participation, coverage and programme sustainability
14. Malecela et al., 2009 [43]/ Tanzania/ 2000–2009	LF elimination programme report	National	To report on the progress made by the Tanzania LF elimination programme.	Establishment of morbidity management programme helped to alleviate patient suffering, reduce social stigma and community support for MDA for LF.	Community participation and programme sustainability
15. Mohammed et al., 2006 [40]/Tanzania (Zanzibar)/2001–2006	LF elimination programme report	National	To highlight the progress of a national LF programme and identify the components required to ensure success through the phases of conception, resource mobilization, Implementation and monitoring.	Components to ensure success of MDA for LF: Mobilize interest from non-governmental development organizations (partnership approaches). Identify the need for morbidity control implementation programme (Including policy on hydrocele surgery). Establish system for monitoring adverse events.	Successful implementation, programme sustainability and community participation
16. Wamae et al., 2006 [41]/ Kenya/Not stated	Mixed methods study	360 households were sampled, with 720 persons interviewed. 65 semi-structured interviews with CDDs, health workers and key informants; and 14 FGDs. (Rural setting) Kilifi and Malindi	To compare the effectiveness of a drug delivery strategy based on mass-treatment by the regular health service with that of community-directed with health system involvement at the implementation stage only.	Community directed treatment + health services arm of the study achieved higher MDA for LF treatment coverage of 88%, compared to the health systems arm which recorded 46.5%.	Coverage, community participation and Programme sustainability
17. Hopkins et al., 2002 [42]/Nigeria/March 2000	Integrated NTD control programme report (pilot)-	Sample was not stated (Rural setting) Plateau and Nasarawa states.	To report on a collaborative effort by the Ministries of Health of Plateau and Nasarawa States, the Federal Ministry of Health and The Carter Center to incorporate health education and Treatment for LF elimination and SH control into ongoing Onchocerciasis activities.	Knowledge Attitudes and Practices (KAP) Survey, a foundation for preparing Health education materials. Integration of LF and SH initiatives within the established onchocerciasis programmes strengthened the latter’s sustainability by capitalizing on cost savings and broadening the program benefits and popularity.	Community participation programme sustainability
18. Gyapong et al., 2001 [34]/Ghana/Not stated	Mixed method study	810 households were interviewed for the quantitative data. Qualitative sample size not clearly stated. (Rural setting) Builsa, Kassena and Nankana Districts.	To Compare the effectiveness of a delivery strategy based on mass-treatment by the regular health-care system with that of a system of community directed treatment only involving the health services at the level of implementation	Health staff and the target communities appreciated the community directed treatment + health services (ComDT/HS) approach more than the health services (HST) stand-alone approach, and were more willing to participate in the community-directed scheme. Method used to distribute the drugs had a marked effect on coverage. The treatment coverage achieved by ComDT/HS (74.5%) was much higher than that of HST (43.5%) The ComDT/HS approach was recommended, especially for areas where access to health facilities is poor and the health workers are over-stretched	Community participation, programmes sustainability and coverage.

**Table 2:** Summary of identified facilitators and barriers

Structural or broad themes	Emergent themes (number of studies)	
	Facilitators	Barriers
Social mobilization/Community engagement/(Health education).	Awareness creation through community led health education programmes (*n* = 3) [36, 37, 42].	Limited investment in appropriate timing, dissemination of accurate MDA for LF information (*n* = 3) [21, 38, 44].
	Innovative and locally relevant means to conduct health education/modern and traditional approaches to H.E. (*n* = 4) [22, 36, 37, 39].	
	Use of appropriate IEC materials for health education (*n* = 5) [22, 24, 36, 37, 42].	
	Involve key health systems representatives and local leaders in health education (*n* = 7) [22, 23, 25, 34, 36, 37, 41]	
Community drug distributors in MDA for LF implementation.	Selection, training and financial incentives provided to CDDs (*n* = 5) [22, 25, 34, 35, 37, 44], and provision of mobile phones and other forms of motivation (*n* = 1) [45].	Limited number of CDDs to implement MDA for LF (*n* = 4) [22, 23, 25, 35].
		Allocation of large number of household areas to CDDs for drug distribution (*n* = 4) [21, 35, 38, 44].
Political and health systems factors in MDA for LF implementation.	Building of partnerships and collaborations (international and local), resulting in sustained political commitment to MDA for LF (*n* = 7) [22, 23, 36, 37, 40, 42, 43].	Major disease outbreaks may paralyze health systems and affect MDA for LF (*n* = 2) [33, 44]
	Integration with existing health interventions (*n* = 4) [36, 37, 39, 42]	
	Innovative resource mobilization strategies in environments totally lacking local resources (*n* = 1) [36]	
	Establishment of morbidity management programmes (*n* = 3) [39, 40, 43]	
	Adverse effects management during MDA for LF implementation (*n* = 6) [34, 36, 37, 39–41].	
Population dynamics affecting MDA for LF Implementation.		Lack of clear geographical demarcations in MDA for LF implementation units (*n* = 2) [22, 23]
		Rapid urbanization and employment seeking population migrations into MDA for LF implementation units (*n* = 2) [22, 23]
MDA for LF drug commodities and logistics supply.		Late delivery and procurement of MDA for LF drugs at community and international level (*n* = 3) [23, 36, 37].
		Unsustainable and inappropriate drug delivery strategies for given settings (*n* = 4) [21, 23, 36, 37].

**Table 3:** Common approaches to improving MDA for LF

Region in SSA	Country of Publication	Common approaches (number of publications)
West-Africa	Mali and Nigeria	Awareness creation through community led health education (H.E) programmes (*n* = 3) [36, 37, 42].
	Mali, Nigeria, Sierra Leone and Togo	Innovative and locally relevant means to conduct health education/modern and traditional approaches to H.E. (*n* = 4) [22, 36, 37, 39].
	Mali, Nigeria and Sierra Leone	Appropriate IEC materials for health education (*n* = 5) [22, 24, 36, 37, 42].
	Mali, Nigeria and Togo	Integration with existing health Interventions (*n* = 4) [36, 37, 39, 42].
West and East Africa	Mali, Nigeria, Sierra Leone Tanzania and Togo	Building of partnerships and collaborations (International and local (*n* = 7) [22, 23, 36, 37, 40, 42, 43].
West and East Africa	Tanzania and Togo	Establishment of morbidity Management programmes (*n* = 3) [39, 40, 43].
West and East Africa	Tanzania, Togo, Sierra Leone, Nigeria, Mali and Ghana	Establishment of adverse effect management Programmes (*n* = 6) [34, 36, 37, 39, 40, 42].
West and East Africa	Ghana, Kenya, Mali, Nigeria, Sierra Leone and Tanzania	Involvement of key health systems representatives and local leaders in health education. (*n* = 7) [22, 23, 25, 34, 36, 37, 41].
West and East Africa	Ghana, Kenya, Mali, Sierra Leone and Tanzania.	Selection, training and financial incentives provided to CDDs (*n* = 5) [22, 25, 34, 35, 37, 45], and provision of mobile phones and other forms of motivation [45] (*n* = 1)

### Factors that facilitate implementation of mass drug administration for lymphatic filariasis

1. **Creating awareness through community health education programmes**
  Maintaining awareness through local health education programmes about the disease and treatment facilitated implementation of MDA for LF. Knowledge about the disease, purpose and benefits of treatment determined the levels of community participation [25, 33–36]. Health workers, community drug distributors (CDDs) and institutions such as churches, mosques, schools and health centers played an important role in driving the local LF health education agenda [21, 25, 34–38]. At the center of all successful MDA for LF campaigns were community led LF behavioral change communication strategies aimed at reaching all people in implementation areas, regardless of their social status. For example, Mali and Nigeria’s integrated neglected tropical diseases (NTDs) control programmes had developed and harmonized disease specific health education messages every year, through community led health education programmes [36, 37]. These community led programmes ensured successful community mobilization and participation.
2. **Engagement of key health systems representatives and local leaders in health education**
  Sustained political commitment from various government departments at both district and national levels towards community health education programmes was cardinal for the success of MDA for LF implementation [37, 39, 40]. Advocacy meetings with health systems representatives at local levels helped to facilitate MDA for LF programme implementation, by bringing these key stakeholders on board and encouraging them to be agents for change in their institutions [22, 23, 25, 34, 36, 37]. Use of traditional leadership and community structures for health education programmes in rural areas was essential for achieving maximum community participation [22, 25, 34]. Involving the community and local structures in MDA for LF programme implementation activities created a sense of ownership by the communities resulting in higher levels of participation.
3. **Innovative and locally relevant means to conduct health education**
  The use of innovative, locally relevant and context specific strategies by community led health education programmes facilitated implementation of MDA for LF [22, 23, 36, 37]. A study from Sierra Leone showed that use of innovative and more “modern” sensitization approaches such as the recurrent dissemination of information on frequently asked questions (FAQs), community radio stations (as platforms for phone-ins, text messaging and chatting with LF experts) as well as use of social media, enabled the reaching of individuals and institutions that had otherwise been unaware of MDA for LF [22]. These innovative approaches provided for better understanding of community concerns, beliefs and potential challenges during the campaigns, which needed to be addressed to achieve maximum community participation. Other examples are the Nigerian, Malian and Togolese programmes where the local media was extensively engaged throughout the MDA for LF campaigns to provide information to the communities, receive feedback and thus make necessary adjustments to the communication strategy [36, 37, 39].
4. **Appropriate information education and communication (IEC) materials for health education**
  Provision and use of appropriate IEC materials was key to successful MDA for LF campaigns’ health education efforts [22, 24, 34, 37, 40, 41]. One study from Sierra Leone highlighted the use of ‘MDA for LF poster images [22],’ which were specifically designed and disseminated in various locations, with information on the treatment protocol and MDA for LF benefits. These IEC materials were developed with input from communities to ensure appropriate, consistent and culturally sensitive information was disseminated. Two Nigerian studies further reported that conducting knowledge attitude and practices (KAP) surveys enabled the LF programme to design target specific, responsive and widely accepted IEC materials [36, 42]. Other benefits included being able to incorporate local understandings and terminologies of LF related conditions on the IEC materials.
5. **Partnerships and collaborations**
  Partnerships and collaborations were essential for sustained and successful implementation of MDA for LF [22, 23, 36, 37, 40, 42, 43]. One of the main reasons for a successful MDA for LF programme in Togo, despite limited resources and political challenges, was formation of strategic international partnerships with institutions like the United States’ Center for Disease Control and Prevention (CDC), Health Development International (HDI) and the Department for International Development (DfID) [39]. These institutions provided funding and technical support for NTD research and the setting up of the lymphedema management programme [39]. Local collaborations with the African Programme for Onchocerciasis Control (APOC) and the Onchocerciasis Control Programme (OCP) also facilitated MDA for LF implementation [36, 37, 42]. Countries that collaborated with both APOC and the OCP recorded considerable reduction in drug distribution costs and sustained high MDA for LF coverage, as both institutions had already established drug distribution structures through community directed treatment (CDT) programmes for onchocerciasis that had been running for many years.
6. **Integration with existing health care interventions**
  Programme integration at national and primary healthcare levels facilitated implementation of MDA for LF [36, 37, 39, 42]. Other NTD programmes such as onchocerciasis, schistosomiasis and trachoma were some of the programmes integrated with MDA for LF. Integration provided a platform for shared coordination and distribution of programme resources as well as harmonization of incentive packages for CDDs across programmes [36, 37, 42]. Furthermore, it facilitated the use and implementation of multiple drug delivery strategies to maximize coverage amongst endemic populations. It also had a considerable impact of reducing the costs of implementing MDA for LF as similar activities could be conducted simultaneously as opposed to separating them. Stand-alone programmes were more costly and difficult to sustain in resource-constrained settings.
7. **Selection and training**
  Adequate training of both the CDDs and healthcare workers prior to MDA for LF campaigns facilitated implementation [22, 25, 34, 35, 37, 44]. Training of CDDs on good communication skills, the disease and its prevention were documented to be important for the CDDs’ acceptance by communities and also for their own motivation in conducting MDA for LF. In Kenya, it was pointed out that attending of workshops and training on mobilization techniques by CDDs not only equipped them with knowledge, but also motivated them to confidently respond to the often challenging LF questions from community members [35]. Similarly, the Togo MDA for LF programme was highly successful due to motivated and well trained CDDs [39], who had undergone similar training with the health workers. The selection process of CDDs, their standing in society and the level of education influenced community participation in MDA for LF [25, 34, 36, 37].
8. **Provision of incentives**
  Provision of appropriate incentives to the CDDs was an essential component of MDA for LF programme success [22, 23, 25, 34, 35]. Incentives differed according to setting. For urban settings, financial incentives were much preferred whilst in the rural setting; villages provided various incentives such as T-shirts, certificates, farmland, levies waiver and above all high social status in the community for the CDDs [21, 35]. In Tanzania, the CDDs indicated better capacity to provide real-time data for MDA programme planning after being provided with mobile phones [45]. In Sierra Leone and Mali, high MDA for LF coverage was attained in urban settings after having previously recorded low coverage by hiring of paid CDDs [22, 23, 37]. These studies established that the volunteer system of CDDs was ineffective in urban settings, especially in over-populated, rapidly urbanizing and mixed ethnic communities.
9. **Management of adverse/side effects**
  Side effects were some of the major reasons why community members did not participate in MDA for LF [21, 38, 40]. Successful MDA programmes had well-established plans for any adverse effects during implementation [34, 36, 37, 39–41]. Qualified healthcare personnel were mandated to not only supervise the CCDs, but also manage on-site, any side effects arising from taking the drugs. Some of the common side effects were nausea, headache, dizziness, fever, malaise, decreased appetite and vomiting. A key component of managing these adverse effects also involved incorporating the messages on side effects into the entire health education package, while underlining that the effects were short term and not clinically harmful. Due to many cultural myths and rumors, it was essential that recipients of these drugs were made aware of the side effects and possible access to care [21].
10. **Establishment of morbidity management programmes**
  Morbidity management programmes for lymphedema and hydrocele were reported to increase community support for and hence participation in MDA for LF [39, 40, 43]. These programmes provided training on self-management of lymphedema for patients and hydrocele surgical operation for the healthcare providers. Community knowledge of available care, including surgery for hydrocele patients motivated people to participate in MDA for LF. Lymphedema management programmes also provided patients with a platform to share information with other community members about the disease and the benefits of the drugs. In Togo and the island of Zanzibar (Tanzania), Lymphedema management programmes helped to maintain community support for MDA for LF through addressing the needs of the individuals in the community with the most visible LF manifestations and providing information about the disease to the family members [39, 40].

### Barriers to implementation of mass drug administration for lymphatic filariasis

1. **Delays in drug delivery and inappropriate strategies**
  Implementation was hindered by the late delivery of drugs at both country and community levels [21, 23, 36, 37]. Delayed shipment of the drugs meant that MDA for LF implementation had to be postponed. Studies from Mali and Sierra Leone reported late deliveries of Ivermectin at country level, which subsequently affected the planning and implementation of MDA for LF [23, 37]. The most preferred drug delivery strategy across all the studies was house-to-house. However, this was unsuitable for urban settings, as people were more mobile with rather different housing arrangements [22, 23, 25]. Four studies from Mali, Kenya and Sierra Leone attained high coverage in urban areas through delivering at central locations such as clinics, schools, hospitals, churches and the use of the street-by-street delivery strategies [22, 23, 25, 37]. Central distribution strategies were reported to be ideal for urban settings, but nonetheless required a lot of effort from the distributors in dealing with large populations as well as managing logistics [36, 37, 39].
2. **Lack of clear geographical demarcations and migrations**
  The lack of clear geographical demarcations and unregistered migrations of indigenous people into rapidly urbanizing settlements hampered the implementation of MDAs for LF [22, 23]. The effect was such that MDA for LF programmes failed to adequately implement because they could not plan for an unknown number of people. In Sierra Leone and Liberia people migrated into cities after the end of the wars in pursuit of better economic opportunities [22, 23, 46]. Such rapidly urbanizing cities became hard to reach and persistently recorded low MDA for LF coverage levels due to these large population movements. Implementation of MDA for LF activities in these rapidly urbanizing and un-demarcated sites was challenging because of the lack of clear boundaries for catchment areas, lack of community identities and health committees to facilitate community engagement and participation [22, 23, 46].
3. **Occurrence of disease outbreaks**
  Disease outbreaks such as the Ebola virus disease (EVD) in West Africa negatively affected MDA for LF programmes. Not only did this mean a shift in national health systems priorities regarding funding, research and development, but also resulted in a temporary halt to all MDA for LF activities in certain areas. In one Liberian study, it was suggested that participation of communities in future MDA for LF would be difficult as people were afraid that either the CDDs or the MDA drugs would spread the deadly Ebola virus disease [33]. Additionally, a Tanzanian study indicated that communities tended to be more concerned about diseases that caused high mortality in the community hence participation in MDA for LF was not such a priority, as they did not feel threatened by the disease [44].
4. **Limited number of community drug distributors**
  CDDs are the frontline personnel in MDA for LF programmes and hence any shortages could negatively affect implementation. Two studies from Mali and Nigeria reported that due to better incentives provided by other well-funded programmes, the capacity to retain CDDs was severely affected [36, 37]. In Mali, it was reported that motivation of CDDs without financial incentives had become a challenge whilst other programmes like those for HIV/AIDs, Malaria and TB were paying them. Similarly, two studies from Sierra Leone indicated that MDA for LF was severely affected during one season when it was concurrently implemented with other primary health care programmes, as the CDDs preferred to work for the other better rewarding programmes [22, 23].
5. **Allocation of large number of households for treatment**
  The allocation of a large number of households for drug distribution to CDDs in limited time periods negatively affected MDA for LF implementation [25, 34, 35]. For example, in one Kenyan study, the CDDs coming from a site that had recorded low MDA for LF coverage complained of their inability to effectively distribute the drugs and conduct health education due to the vastness of the areas and the limited time period allocated to the exercise. They indicated that they had failed to cover all the allocated households and could not make any call backs to attend to people who had missed the actual drug distribution days [35]. The large number of households also had a bearing on the CDDs capacity to complete timely distributions and report accurate treatments figures on the tally sheets. Three studies from Kenya, Tanzania and Ghana further indicated that community members did not participate in one MDA for LF campaign because they had not been reached by the CDDs [21, 24, 38].

### Summary of the common approaches to improve MDA for LF implementation

Publications that discussed approaches aimed at improving MDA for LF implementation were further analyzed and categorized by country and region. We identified that innovative approaches to social mobilization through community led health education programmes and integration with existing health interventions were mostly documented in the West African region. Partnership approaches, morbidity management, adverse effects management and incentives for CDDs were crosscutting between the West and East African region (Table 3).

## Discussion

The main factors facilitating implementation of MDA for LF programmes were awareness creation through innovative community health education programmes, creation of partnerships and collaborations, integration with existing NTD programmes, motivation of CDDs through appropriate incentives and training mechanisms, management of adverse effects and creation of morbidity management programmes. Some of the major barriers to implementation included the lack of geographical demarcations and unregistered migrations into rapidly urbanizing areas, major disease outbreaks like the Ebola virus disease in West Africa, delayed drug deliveries at both country and community levels, inappropriate drug delivery strategies, limited number of CDDs and allocation of large number of households for drug distribution.

Awareness creation efforts involving the use of innovative and socially appropriate health education or behavioral change messages informed by KAP surveys can facilitate implementation of future MDAs for LF. Conducting KAP surveys prior to MDA for LF implementation helps to minimize misinformation by developing standardized messages and IEC materials that address community concerns. Several other studies have highlighted the importance of locally relevant community health education initiatives in MDAs for LF [47–49]. Krentel et al.’s [50] systematic review of factors that influence individual compliance to MDA for LF also emphasizes the need to adapt community health education strategies to local contexts.

Strategic partnerships and collaborations are essential for successful implementation of MDA for LF programmes because they leverage the limited government resources and guarantee sustained political commitment from local authorities. Bush et al [8], highlights the role of partnerships in MDA for LF and other NTD control activities by stating that “partners advocate and facilitate progress in operational research, programmatic development, capacity building, resource mobilization and monitoring.” Furthermore, community partnerships provide a platform to build respectful relationships, engender trust and sustain community support towards MDA for LF programmes [51].

Though integration of MDA for LF with other interventions was found to facilitate implementation, the different drug requirement frequencies, as well as time lengths and target areas make it a complex process. Various concerns regarding integration of MDAs have been raised including possible side effects from co-administration of drugs, and challenges in monitoring and evaluation [52]. One study found that integrating MDA for LF with onchocerciasis was highly achievable, but much more complicated when done with schistosomiasis [53].

To be effective, integration requires careful consideration of several issues including the geography, epidemiology and ecology of different NTDs, in addition to the advantages and disadvantages of existing control strategies [54]. It is important that any integration efforts are aimed at strengthening health systems and developed within primary healthcare, to encourage programme continuity and sustainability [55]. Additionally, where two or more programmes cannot be fully integrated, it is certainly possible that co-planning allows the programmes to move forward at their separate time frames, as other programmatic activities can still be coordinated.

Delays in drug delivery at national and community levels suggest the need for proactive strategic planning from programme implementers to tackle unforeseen drug shipment delays and logistical challenges encountered by the CDDs. The intricate nature of the work that CDDs perform in MDAs for LF demands for consistent motivation. Several motivating factors have been suggested by Njomo et al., that include provision of transportation, capacitation and training, proper supervision, trust and familiarity with community and recognition [35].

Occurrence of major disease outbreaks like the Ebola virus disease in West Africa may interrupt MDA for LF activities, hence the need to adequately prepare for such kind of eventualities [33, 46]. MDA for LF programmes may not only be interrupted, but possible evolution of negative cultural sentiments towards MDA drugs may occur due to the nature of the disease outbreak. This entails the need for LF MDA programmes to reinforce health education campaigns aimed at tackling negative community sentiments.

Deficiency of definite information about populations at risk in rapidly urbanizing un-demarcated areas that experience high-unregistered migrations, affects the quality of monitoring and evaluation, coverage estimation and the planning of treatment supplies. Lack of demarcations also negatively affects the mapping of the geographical distribution of LF disease. Indeed, rapid and long-term migrations may affect the disease epidemiology hence hindering MDA for LF activities. Some authors have suggested the need to monitor population dynamics when planning MDA for LF [56, 57].

Reducing the challenges to the implementation process of MDA for LF would require adopting a system thinking approach. This approach may be relevant because it demands careful consideration of possible consequences of various interventions through team work and collaborative thinking by relevant stakeholders [58]. The involvement of various stakeholders would help to critically consider in an iterative and systematic manner, the interactions between MDA for LF and other components within the local health systems [59]. Key health systems components which could be considered include resources (health workers, finances, drugs, and information), health service delivery systems, governance or leadership as well as community norms and values [60].

Reaching the 2020 global target for LF elimination will require multi-sectoral approaches and integration of the already effective control strategies. This will mean not only focusing our attention on MDA for LF, but also strengthening vector control strategies and compliance to mosquito net use in endemic areas. Furthermore, MDA for LF implementation teams should systematically consider the factors that have been outlined in this review, determine their relevance to the local context and develop a plan to specifically address these issues in advance of the implementation efforts. Formative research should be undertaken to focus on any specific or additional contextual issues, where the generated information would be valuable for good implementation. For example, the 7 facilitating factors and the 5 barriers could be assigned to specific team members to assess, determine and record how these factors will be addressed prior to implementation.

### Study strengths and limitations

One of the major limitations of this review is the paucity of SSA literature explicitly discussing ‘MDA for LF implementation’ as most articles were focused on reporting about the disease prevalence. Lack of publications from the francophone region in SSA is another limitation to our review. However, we tried to mitigate this by including two studies from Togo and Mali, and we also triangulated our findings with other robust systematic reviews like Krentel et al.’s [50]. One of the key strengths in this review lies in the extra effort to extensively search the literature from different countries in SSA. Another strength is the inclusion of papers with a wide methodological variety, allowing us to capture a wide range of issues surrounding implementation of MDA for LF.

## Conclusion

This systematic review has highlighted various factors that shape implementation of MDA for LF. Key areas of success that should be considered for every successful MDA for LF undertaking include those facilitating the implementation process such as; building of strategic partnerships for innovative resource mobilization, especially in resource-limited settings, exploring possibilities of programme integration both at national and primary healthcare levels and extensive engagement of the community in programme implementation efforts. Logistical, geographical and biological barriers to MDA for LF implementation need careful consideration before programme design and implementation.

The need to understand context specific factors shaping implementation of MDA for LF is not only important for SSA, but also for other countries at risk of infection. This understanding will form the basis for all planning, organization and implementation of MDA for LF, if we are to reach the WHO 2020 target of elimination. We therefore recommend that research on MDA for LF not only focuses on drug delivery and uptake, but more so on the main implementation issues as identified by the review. Further research should explore in detail the different approaches taken to improve MDA for LF implementation by employing some of the general steps used in models of implementation research.

## Abbreviations

APOC: African Programme for Onchocerciasis Control
CDDs: Community drug distributors
CHVs: Community health volunteers
DEC: Diethylcarbamazine citrate
EVD: Ebola virus disease
FAQs: Frequently asked questions
GPELF: Global Programme to Eliminate Lymphatic Filariasis
HE: Health education
KAP: Knowledge attitudes and practices
LF: Lymphatic filariasis
MDA for LF: Mass Drug Administration for Lymphatic Filariasis
NTDs: Neglected Tropical Diseases
OCP: Onchocerciasis Control Programme
